# Kinetics of vacancy annealing upon time-linear heating applied to dilatometry

**DOI:** 10.1007/s10853-017-1780-4

**Published:** 2017-11-09

**Authors:** R. Enzinger, Chr. Neubauer, J. Kotzurek, W. Sprengel, R. Würschum

**Affiliations:** Institute of Materials Physics, Graz University of Technology, Petersgasse 16, 8010 Graz, Austria

## Abstract

A kinetic model for the diffusion-controlled annealing of excess vacancies under the experimentally relevant, non-isothermal condition of time-linear heating is presented and applied to dilatometry. The evolution of the vacancy concentration with time is quantitatively analyzed, considering as ideal sinks either dislocations or grain boundaries of spherical- or cylindrical-shaped crystallites. The validity of the model is tested using dilatometry data that were obtained for ultrafine-grained Ni prepared by high-pressure torsion. The entire two-stage annealing curve of the dilatometric length change can be analyzed by combining the present kinetic model of vacancy annealing at grain boundaries with established non-isothermal kinetics of recrystallization.

## Introduction

Dilatometry as a powerful thermo-analytical tool has recently been more and more applied to study absolute concentrations and kinetics of defects in metals [1]. The basic idea is that upon annealing-induced removal of defects which are associated with excess volume, such as vacancies, dislocations, or grain boundaries, a macroscopic shrinkage of the solid occurs in reference to a defect-free sample. In this way, the absolute concentration of lattice vacancies as well as the excess volume of grain boundaries could be determined for ultrafine-grained metals prepared by techniques of severe plastic deformation, i.e., high-pressure torsion (HPT) and equal-angular channel pressing [2–5]. Similar to differential scanning calorimetry (DSC), these measurements are often performed in the mode of time-linear heating rather than isothermally due to reasons of signal stability and strength. Moreover, various annealing processes can be scanned in one single run which is of relevance since the processes are inherently irreversible so that each sample can be used only once.

Different defect types may be distinguished by their distinct temperature regimes of annealing during linear heating which is reflected in more or less well-separated annealing stages of the irreversible length change curve $\Delta l/l (T)$. For the length change associated with the annealing of grain boundaries in the wake of crystallite growth, a kinetic model for non-isothermal heating already exists on the basis of the Johnson–Mehl–Avrami–Kolmogorov theory (JMAK) ([6, 7], see below, “Recrystallisation” section) which was applied to dilatometry data on ultrafine-grained nickel by Oberdorfer et al. [8].

The present work aims to extend non-isothermal modeling to point defects as well, by developing a kinetic model for the diffusion-controlled annealing of excess vacancies at various types of sinks under the non-isothermal condition of time-linear heating. For ultrafine-grained crystallites, major sinks of vacancies are grain boundaries. Here, spherical- and cylindrical-shaped grains will be considered (“Annealing of vacancies at grain boundaries” section), geometries which have earlier been treated for thermal analysis of evaporation of toluene from an epoxy resin by Ozawa [9]. A major type of sink in single or coarser-grained crystals is dislocations. Following Seidmann and Balluffi [10] dealing with dislocations as sources of thermally generated vacancies, in the present work the annealing of vacancies for straight dislocations is modeled (“Annealing of vacancies at dislocations” section). In the discussion (“Discussion” section), the model is tested using ultrafine-grained HPT-deformed Ni as a case study and the model is compared to other models of vacancy kinetics in metals [11].

## Modeling of annealing kinetics

In the following, kinetic models are presented for the non-isothermal annealing of lattice vacancies at dislocations (“Annealing of vacancies at dislocations” section) and at grain boundaries of spherical- or cylindrical-shaped crystallites in metals (“Annealing of vacancies at grain boundaries” section). For the sake of comparison, the kinetics of non-isothermal recrystallization is summarized in “Recrystallisation” section.

### Annealing of vacancies at dislocations

The annealing kinetics of vacancies at dislocations is modeled assuming a regular array of parallel straight dislocation lines. The straight dislocation is considered as cylinder (radius *a*), the surface of which acts as ideal sink. Lattice vacancies within a concentrical hollow cylinder around the dislocation anneal out by diffusion toward the inner wall at the center with the cutoff radius *a* of the dislocation. The outer radius *b* of the hollow cylinder is related to the dislocation density $N_\mathrm{d}$ per unit area by

1 $$\begin{aligned} b = \frac{1}{\sqrt{\pi N_\mathrm{d}}} \ . \end{aligned}$$

Due to cylindrical symmetry, the diffusion equation for the vacancy concentration *C*(*r*, *t*) reads:

2 $$\begin{aligned} \frac{\partial C}{\partial t} = D \, \Big (\frac{\partial ^2 C}{\partial r^2} \, + \, \frac{1}{r} \, \frac{\partial C}{\partial r}\Big ), \quad a< r < b \end{aligned}$$

where *D* denotes the vacancy diffusion coefficient, *r* the radius, and *t* the time.

For taking into account non-isothermal conditions and, therefore, a time-dependent diffusion coefficient *D*(*t*), we use the ansatz

3 $$\begin{aligned} C(r, t) = U(r)\cdot X(t) \,. \end{aligned}$$

With the separation constant $-\beta ^2$, this leads to the time-dependent part

4 $$\begin{aligned} \frac{\mathrm{d} X}{X} = -\beta ^2 D(t) \mathrm{d}t \, . \end{aligned}$$

For the usual case of time-independent *D*, the solution of Eq. (4) reads (see, e.g., the textbook of Carslaw and Jaeger [12]):

5 $$\begin{aligned} X(t) = \exp \big (-D \, \beta ^2 \, t\big ) \, . \end{aligned}$$

For the spacial part *U*(*r*), the Bessel differential equation of zero order is obtained from Eq. (3):

6 $$\begin{aligned} \frac{\mathrm{d}^2 U}{\mathrm{d}r^2} \, + \, \frac{1}{r} \frac{\mathrm{d}U}{\mathrm{d}r} \, + \, \beta ^2 U = 0 . \end{aligned}$$

The ideal sink behavior of the dislocation gives the boundary condition at the inner cutoff radius:

7 $$\begin{aligned} U(r=a) = 0 \ . \end{aligned}$$

The outer boundary condition

8 $$\begin{aligned} \left. {} \frac{\partial U}{\partial r} \right| _{r\,=\,b} = 0 \end{aligned}$$

reflects the vanishing vacancy flux through the outer border ($r = b$) of the diffusion cylinder. The solution of Eq. (6) satisfying the inner boundary condition (Eq. 7) is given by [12]

9 $$\begin{aligned} U(\beta r) = Y_0(\beta a) J_0(\beta r) - J_0(\beta a) Y_0(\beta r) \end{aligned}$$

where $J_0$ and $Y_0$ denote Bessel functions of the first and second kind, respectively. The outer boundary condition (Eq. 8) yields a conditional equation for $\beta$

10 $$\begin{aligned} -\,J_1(\beta b) Y_0(\beta a) + J_0(\beta a) Y_1(\beta b) = 0 \end{aligned}$$

that is solved by a set of discrete values $\beta = \beta _n$.

With $U(\beta _n r)$ representing the solution of Eq. (6) for $\beta = \beta _n$, a partial solution of the diffusion Eq. (2) reads $\exp (-D \, \beta _n^2 \, t) \cdot U(\beta _n r)$. Due to the linearity of the diffusion equation, the general solution is given by the superposition of the partial solutions for different $\beta _n$:

11 $$\begin{aligned} C(r,t) = \sum _{n=1}^{\infty } A_n \, \exp \big (-D \, \beta _n^2 \, t \big ) \, U(\beta _n r). \end{aligned}$$

The coefficients

12 $$\begin{aligned} A_n = \frac{\int _a^b{r \, C(r,0) \, U(\beta _n r) \ \mathrm{d}r}}{\int _a^b{r \, U^2(\beta _n r) \ \mathrm{d}r}} = \frac{N(\beta _n)}{T(\beta _n)} \end{aligned}$$

are to be determined by the initial condition *C*(*r*, 0).

Assuming a homogeneous initial distribution $C(r,0) = C_0$ of lattice vacancies, the numerator $N(\beta _n)$ and denominator $T(\beta _n)$ of $A_n$ (Eq. 12) can be calculated. Taking into account properties of the Bessel functions ([13], see Appendix) and making use of Eq. (10), one obtains for the numerator

13 $$\begin{aligned} N(\beta _n) = -\displaystyle { \frac{2 C_0}{\pi \beta _n^2} } \end{aligned}$$

and after some algebra for the denominator

14 $$\begin{aligned} T(\beta _n) =&\,\displaystyle {\frac{b^2}{2}} \bigl (Y_0(\beta _n a) J_0(\beta _n b) - J_0(\beta _n a) Y_0(\beta _n b) \bigr )^2 \nonumber \\&\quad -\displaystyle {\frac{a^2}{2}} \bigl (Y_0(\beta _n a) J_1(\beta _n a) +J_0(\beta _n a) Y_1(\beta _n a) \bigr )^2 \, . \end{aligned}$$

**Figure 1:**
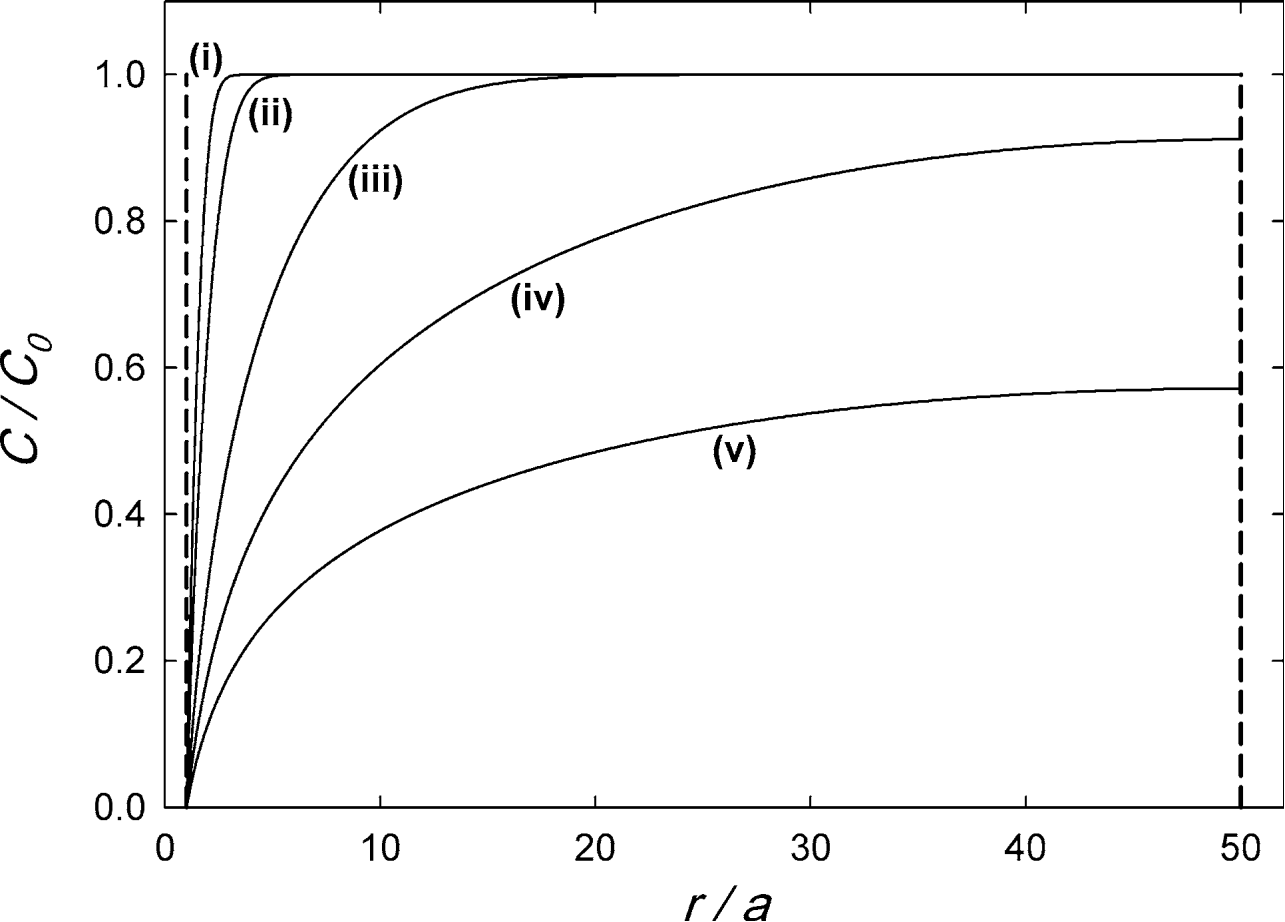
Cylinder-symmetrical vacancy concentration profile *C*(*r*, *t*) for diffusion-controlled vacancy annealing at dislocations in dependence of *r* / *a* with the inner cutoff radius $a= 0.3$ nm and the outer radius of the diffusion cylinder of $b=15$ nm (corresponding to $r/a = 50$). The curves represent different values $D \times t$ showing the evolution of the profile with time *t*. (i): $D \times t = (b/100)^2$, (ii): $(b/50)^2$, (iii): $(b/10)^2$, (iv): $(b/2)^2$, (v): $b^2$

For the sake of illustration, concentration profiles *C*(*r*, *t*) according to the above solution (Eqs. 11, 12, 13, 14) are shown in Fig. 1 for different values of $D \times t$.

Integrating the concentration of vacancies *C*(*r*, *t*) over the total volume of the hollow cylinder yields the fraction $\alpha (t)$ of vacancies not yet annealed out at the time *t*:

15 $$\begin{aligned} \alpha (t) = \int _a^b{ C(r,t) \, 2 \pi r \ \mathrm{d}r} \, . \end{aligned}$$

After insertion of Eq. (11) and integration using the properties of Bessel functions (see “Appendix”) and Eq. (10), one obtains

16 $$\begin{aligned} \alpha (t) = \displaystyle {\sum _{n=1}^{\infty } \frac{8}{\pi \, \beta _n^3 \, T(\beta _n)} \times \exp \big (-D \, \beta _n^2 \, t\big )} \end{aligned}$$

as a function of time *t* with $T(\beta _n)$ according to Eq. (14) and $\beta _n$ denoting the roots of Eq. (10).

Next, we consider the non-isothermal case for which the diffusion coefficient *D* in general becomes time-dependent. For the important experimental situation of time-linear heating with a rate *h*, the thermally activated diffusion coefficient reads:

17 $$\begin{aligned} D(t) = D_0 \ \exp {\Big (-\frac{Q}{k_B \, (T_0 + h \cdot t)}\Big ) }, \end{aligned}$$

where $k_B$ denotes the Boltzmann constant, $T_0$ the starting temperature, *Q* the activation energy of diffusion, and $D_0$ the pre-exponential factor. With $D(\tau )$ according to Eq. (17), the following solution is obtained for the time-dependent part (Eq. 4) of the diffusion equation:

18 $$\begin{aligned} X(t)= & {} \exp \Big (- \beta ^2 \, \int _0^t \! D(\tau ) \, \mathrm {d}\tau \Big ) \nonumber \\= & {} \exp \Big (- \beta ^2 \, \frac{D_0 \, Q}{k_B \, h} \, \Big [\frac{\mathrm {e}^{-x'}}{x'} + \mathrm {Ei} (-\,x') \Big ]_{x_0}^x\Big ) \end{aligned}$$

where $\mathrm {Ei}(x)$ denotes the exponential integral function [13] and

19 $$\begin{aligned} x = x'(\tau = t) =&\displaystyle {\frac{Q}{k_B \, (T_0 + h \cdot t)}} \, , \end{aligned}$$

20 $$\begin{aligned} x_0 = x'(\tau = 0) =&\displaystyle {\frac{Q}{k_B \, T_0}} \,. \end{aligned}$$

Irrespective of the time dependence of *D*, the diffusion Eq. (2) is linear with solutions given by any superposition of partial solutions for different $\beta _n$. Moreover, the time dependence of *D* does not affect the boundary conditions (see Eqs. 7 and 8). Therefore, the root equation for $\beta _n$ (Eq. 10) and the coefficients $A_n$ (Eq. 12) are also valid with Eq. (18), so that for the solution for time-linear heating Eq. (16) modifies to

21 $$\begin{aligned} \alpha (t) = \displaystyle {\sum _{n=1}^{\infty } \frac{8}{\pi \, \beta _n^3 \, T(\beta _n)}} \, \times \displaystyle {\exp \Big (- \beta _n^2 \, \frac{D_0 \, Q}{k_B \, h} \, \Big [\frac{\mathrm {e}^{-x'}}{x'} + \mathrm {Ei} (-x') \Big ]_{x_0}^x \Big )} \end{aligned}$$

with $T(\beta _n)$ and $\beta _n$ according to Eqs. (14) and (10), respectively, as above. We note that the solution differs from that of Seidman and Balluffi [10] due to the different boundary and initial conditions.

### Annealing of vacancies at grain boundaries

In ultrafine-grained materials, even more prevalent than vacancy annealing at dislocations, may be the annealing out of lattice vacancies at grain boundaries. Here, we consider the two important cases of spherical- and cylindrical-shaped crystallites for which the solutions of the diffusion equation for constant diffusion coefficient are given in the textbook of Crank [14]. In analogy to Eq. (16), the fraction $\alpha (t)$ of vacancies not yet annealed out at the time *t* reads for grain boundaries of spherical-shaped crystallites

22 $$\begin{aligned} \alpha (t) = \frac{6}{\pi ^2} \ \sum _{n=1}^{\infty } \ \frac{1}{n^2} \, \exp \Big (\frac{-D \, n^2 \, \pi ^2 \, t}{a^2}\Big ) \end{aligned}$$

and for cylindrical-shaped crystallites

23 $$\begin{aligned} \alpha (t) = \sum _{n=1}^{\infty } \ \frac{4}{a^2 \beta _n^2} \ \exp \Big (-D \, \beta _n^2 \, t\Big ) \end{aligned}$$

where *a* denotes the radius. As in the previous subsection, here the grain boundaries are considered as ideal sinks and a homogeneous initial vacancy distribution in the crystallites is assumed. For the cylindrical symmetry (Eq. 23), $\beta _n$ is determined by the roots of the Bessel function

24 $$\begin{aligned} J_0(a \beta _n) = 0. \end{aligned}$$

This textbook solution can be extended to the case of time-linear heating with time-dependent diffusion coefficient according to Eq. (17). Again, in analogy to Eq. (21), one obtains for the case of spherical crystallites

25 $$\begin{aligned} \alpha (t) = \frac{6}{\pi ^2} \ \sum _{n=1}^{\infty } \ \frac{1}{n^2} \, \exp \left(-\frac{\, n^2 \, \pi ^2}{a^2} \, \cdot \, \frac{D_0 \, Q}{k_B \, h} \, \left[\frac{\mathrm {e}^{-x'}}{x'} + \mathrm {Ei} (-x') \right ]_{x_0}^x  \right) \end{aligned}$$

and for cylindrical crystallites

26 $$\begin{aligned} \alpha (t) = \sum _{n=1}^{\infty } \ \frac{4}{a^2 \beta _n^2} \ \exp \left(-\beta _n^2 \, \frac{D_0 \, Q}{k_B \, h} \, \left[\frac{\mathrm {e}^{-x'}}{x'} + \mathrm {Ei} (-x') \right]_{x_0}^x  \, \right ) \end{aligned}$$

with $\beta _n$ according to Eq. (24). We note that these solutions for spherical and cylindrical geometry in general correspond to those derived by Ozawa [9]; however, the present treatment refrains from an approximation [15] of the exponential integral function as applied earlier [9].

**Figure 2:**
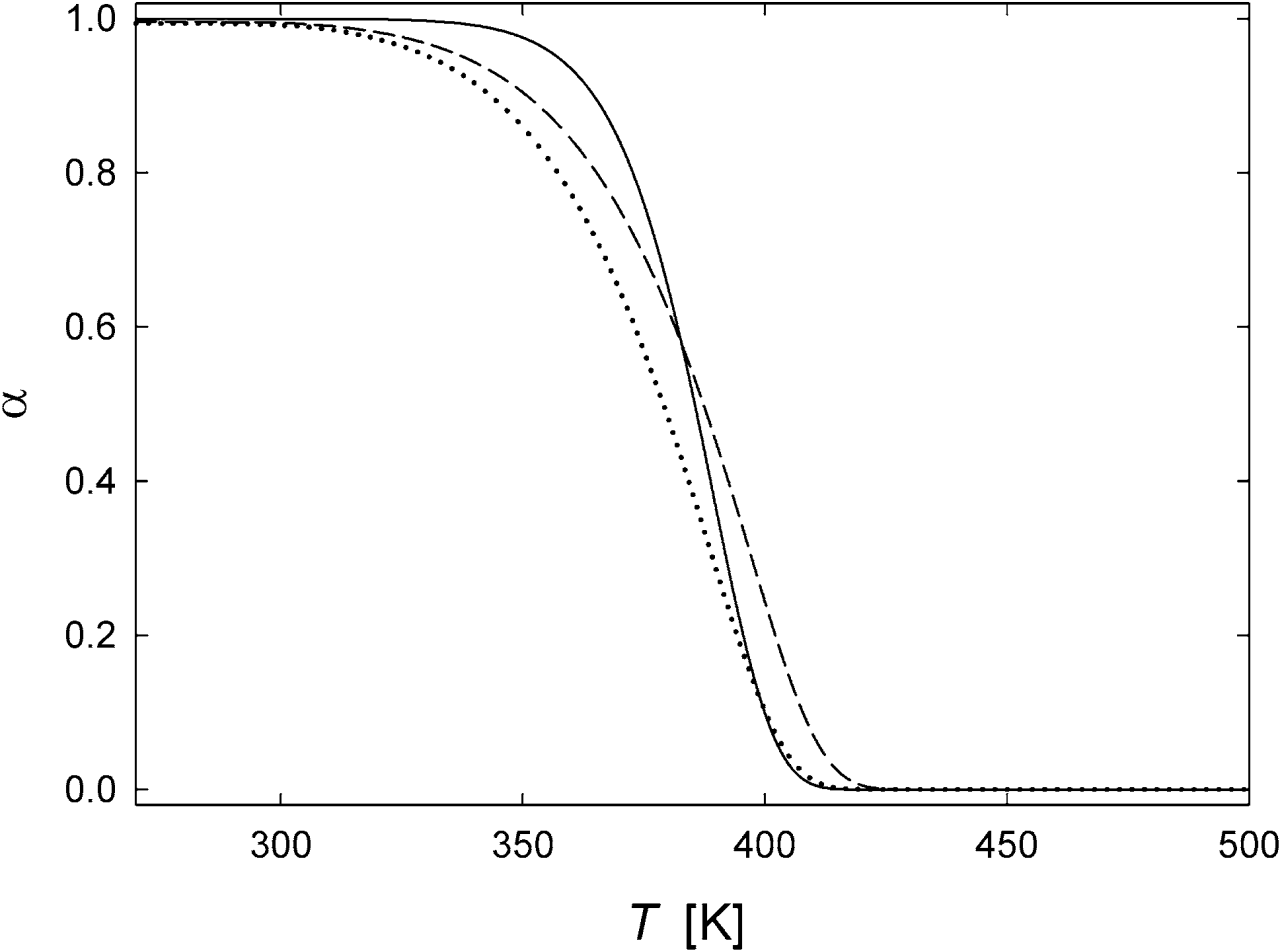
Fraction $\alpha$ of vacancies not yet annealed according to time-linear heating kinetics of diffusion-controlled vacancy annealing at the three types of ideal sinks modeled in this paper: straight dislocations (solid line, Eq. 16), grain boundaries of spherical- (dotted line, Eq. 25) and cylindrical-shaped crystallites (dash-dotted line, Eq. 26). Parameters are as follows: heating rate $h=6$ K/min, pre-exponential factor $D_0 = 9.2\times 10^{-5}$ m$^2$/s, activation energy $Q = 1.04$ eV, radius of cylindrical or spherical crystallites $a = 100$ nm (Eqs. 25 and 26), inner cutoff $a = 0.3$ nm, and outer radius $b =21$ nm for dislocation model Eq. (16)

The computed vacancy annealing curves for the three types of sink geometries are shown in Fig. 2 using for the vacancy diffusion data exemplarily those of nickel according to literature, i.e., a pre-exponential factor $D_0 = 9.2\times 10^{-5}$ m$^2$/s and an activation energy $Q = 1.04$ eV [16]. Here, $a = 100$ nm is assumed for the radii of the cylindrical or spherical crystallites. For dislocations with an inner cutoff radius $a = 0.3$ nm [10], the outer radius of $b =21$ nm is adjusted so that the vacancy annealing at dislocations occurs in the same temperature regime as the annealing at grain boundaries. The value $b =21$ nm corresponds to a dislocation density of $\rho = 7.2\times 10^{14}$ m$^{-2}$ (Eq. 1).

It becomes apparent that in the case of identical crystallite size the vacancy annealing at grain boundaries of spherical-shaped crystallites occurs earlier during time-linear heating compared to cylindrical-shaped crystallites (compare dotted and dashed line in Fig. 2, respectively). This reflects the reduced mean diffusion length for reaching the sinks in the case of spheres compared to cylinders. Comparing the cylindrical-symmetrical cases of vacancy annealing at grain boundaries or dislocations (dashed or full line in Fig. 2, respectively), it is worthwhile to point out that the maximum diffusion radius (here $b = 21$ nm), i.e., the distance between the dislocations, has to be much smaller than the crystallite radius ($a = 100$ nm) for obtaining vacancy annealing in the same regime of heating. This is due to the fact that the narrow inner cylindrical area which acts as sink in the case of the dislocation is much smaller than the outer cylindrical area acting as sink in the case of the grain boundary.

In the example above, a starting temperature $T_0 = 270$ K was used for the lower bound $x_0$ (Eq. 20). Replacing this lower bound by $x_0 \rightarrow \infty$, i.e., setting $T_0 = 0$ K, does not affect the result in this case. One should, however, consider that significant deviations may occur when the annealing stage is only slightly above $T_0$.^1^

### Recrystallisation

In order to compare the kinetic model of vacancy annealing with experimental annealing data on extremely deformed, ultrafine-grained metals, we present for the sake of completeness the non-isothermal solution of recyrstallization kinetics. The Johnson–Mehl–Avrami–Kolmogorov (JMAK) model of recrystallization was first adapted to non-isothermal kinetics by Henderson [6]. Under conditions of time-linear heating with the rate *h*, Louis and Garcia-Cordovilla [7] obtained for the fraction $\alpha$ not yet transformed up to the time *t*:

27 $$\begin{aligned} \alpha (t) = \exp \left[ -\left( \frac{K_0 \, Q}{k_B \, h} \, \int _x^{\infty } \frac{\exp (-x')}{x'^2} \, \mathrm{d}x'\right) ^n \right] \end{aligned}$$

with *x* according to Eq. (19).^2^ *n* denotes the Avrami exponent and *Q* or $K_0$ the activation energy or the pre-exponential factor of the recrystallization rate function $K(T) = K_0 \, \exp (-Q/(k_B T))$, respectively (see also [8]).

To be conformal with the above solutions for vacancy annealing, we replace the upper integration limit by $x_0 = Q/(k_B T_0)$ (see Eq. 20). Similar as in the previous subsections, the integral can be expressed by the exponential integral function, yielding:

28 $$\begin{aligned} \alpha (t) = \exp \left[ - \left( \frac{K_0 \, Q}{k_B \, h} \, \Big [\frac{\mathrm {e}^{-x'}}{x'} + \mathrm {Ei} (-\,x') \Big ]_{x_0}^x \right) ^n \, \right] \, . \end{aligned}$$

As above, using the lower bound $x_0 \rightarrow \infty$ is justified as long as the recrystallization stage lies well above $T_0$.

## Discussion

**Figure 3:**
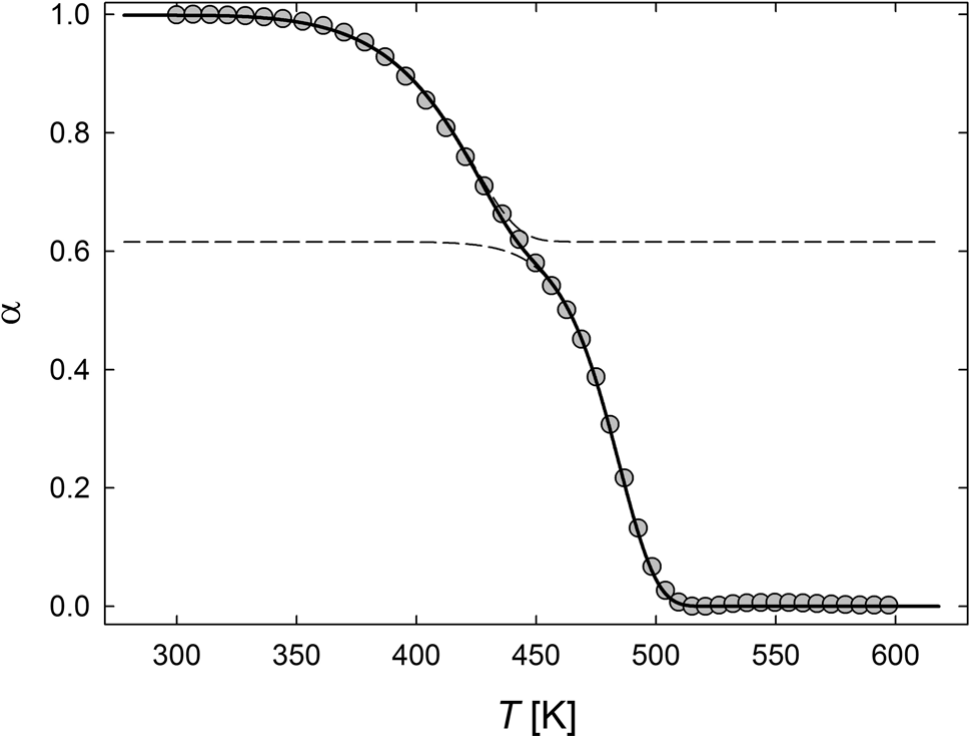
Two-stage dilatometer annealing curve of HPT-Ni in dependence of temperature *T* upon heating at a rate *h* of 6 K/min. Comparison of experimental data (dot) with the combined models of vacancy annealing at GBs of cylindrical crystallites (first stage) and subsequent recrystallization (second stage). $\alpha$: fraction not yet annealed out or transformed (Eqs. 26,28); relative length change $\Delta l/l = 2.7\times 10^{-4}$ for both stages. Model parameter for vacancy stage (Eq. 26): $Q = 1.04$ eV [16], $D_0 = 2.3\times 10^{-5}$ m$^2$/s. Model parameter for recrystallization stage (Eq. 28): $n = 1.1$, $Q = 1.2$ eV, $K_0 = 1.8\times 10^{10}$ 1/s. Common model parameters: relative fraction of vacancy (recrystallization) stage: 0.38 (0.62); mean crystallite radius $a = 100$ nm with a Gaussian distribution of width $\sigma = 25$ nm taking crystallite radii in steps of 5 nm over a width of $2 \sigma$ (i.e., between 75 and 125 nm). Solid line: sum of the two model curves; dashed lines: model curves for each of the two stages

The present model is particularly well suited for quantitatively analyzing dilatometry data of materials prepared by severe plastic deformation, such as high-pressure torsion [17]. These materials may contain a high-abundant concentration of lattice vacancies as well as a high number of grain boundaries associated with the submicron crystallite size. HPT-Ni is an attractive model system, since lattice vacancies in Ni are stable at ambient temperature and become mobile at temperatures around 360 K [16] below the recrystallization stage which starts at about 470 K [2]. Therefore, with respect to the measurement, the length change associated with the annealing out of vacancies is well separated from the length change which occurs due to the removal of grain boundaries (GBs) upon crystallite growth (Fig. 3). Also, the model assumptions apply well for this case since the crystallite size remains constant during diffusion-mediated vacancy annealing and the character of the GB sinks may be considered as stable during this annealing process due to the high sink density.

Figure 3 shows the experimental two-stage variation of the dilatometer length change of HPT-Ni [18] along with an analysis according to the present model. The model represents a superposition of vacancy annealing at GBs with subsequent recrystallization. Since HPT-Ni has an anisotropic structure with elongated grain shape [2, 5], the solution for the cylinder-symmetrical case (Eq. 26) was used for modeling. The crystallite size was deduced from scanning electron microscopy approximating the size distribution by a Gaussian function with the values as quoted in the caption of Fig. 3. Apart from the activation energy of vacancy migration, the model parameters were obtained from fitting the experimental two-stage curve; these parameters are summarized in the figure caption as well. It is apparent from Fig. 3 that the experimental data can be described perfectly by the present model. The relative fractions of both stages are given by the clearly discernible transition between the two substages. The activation energy of 1.2 eV obtained for the recrystallization stage is the same as that deduced earlier from Kissinger analysis of the shift of the stage with the heating rate [8].

Although the presented modeling of vacancy annealing kinetics was stimulated by our experimental studies on HPT metals, the application of this model is by far not restricted to this type of materials. In fact, the model is suitable for other issues of vacancy annealing as well, for instance, for the analysis of annealing of excess vacancies which are obtained by quenching of vacancies formed at high temperatures in thermal equilibrium.

Finally, the presented model on vacancy annealing at different types of sinks will be compared with that reported by Fischer et al. [11]. The model developed by Fischer et al. [11] is based on non-equilibrium Onsager’s thermodynamic [19] and treats the generation and annihilation of vacancies at sources and sinks from the view point of continuum mechanics in a general manner taking into account mechanical driving forces and the role of bulk viscosity. Similar to the present model, ideal behavior of the vacancy sinks (dislocations, grain boundaries) is supposed. Whereas for the general case, the resulting differential equations are to be solved numerically, for isothermal conditions only analytical solutions are derived for vacancy annealing at dislocations and GBs of spherical crystallites from which the time evolution of the vacancy concentration can be determined implicitly.

Compared to ref. [11], the present direct ansatz based on atomistic diffusion yields closed-form expressions right for the practical important non-isothermal case of time-linear heating. In addition to dislocations and GBs of spherical crystallites, also GBs of cylindrical crystallites were considered as sinks. As exemplarily shown for the test case of HPT-Ni, these solutions can be combined with JMAK kinetics of recyrstallization and allow a comparison with experimental data in a straightforward manner. Despite its simplicity, our model is considered to capture the most relevant features of vacancy annealing. It should be pointed out that this model of vacancy annealing is not restricted to dilatometry, but may be applied to other techniques of thermal analysis as well, such as differential scanning calorimetry.

## Appendix

The following formulae for Bessel functions [13] are used in “Annealing of vacancies at dislocations” section

29 $$\begin{aligned} J_1(z) Y_0(z) - J_0(z)Y_1(z)= & {} \frac{2}{\pi z} \end{aligned}$$

30 $$\begin{aligned} \int _a^b{r \, J_0(\beta r) \, \mathrm{d}r}= & {} \frac{1}{\beta } \, \big (b \, J_1(\beta b) \, - \, a \, J_1(\beta a) \big ) \end{aligned}$$

31 $$\begin{aligned} \int _a^b{r \, J_0^2(\beta r) \, \mathrm{d}r}&= \, {} \frac{b^2}{2} \, \Bigl ( \big [J_1(\beta b)\big ]^2 \, + \, \big [J_0(\beta b)\big ]^2 \Bigr ) \, \nonumber \\&\quad - \frac{a^2}{2} \, \Bigr ( \big [J_1(\beta a)\big ]^2 \, + \, \big [J_0(\beta a)\big ]^2 \Bigr ) \end{aligned}$$

32 $$\begin{aligned} \int _a^b{r \, J_0(\beta r) Y_0(\beta r) \, \mathrm{d}r}= & {} \frac{b^2}{2} \, \bigl ( J_0(\beta b) Y_0(\beta b) + J_1(\beta b) Y_1(\beta b) \bigr ) \, \nonumber \\&\quad -\frac{a^2}{2} \, \bigl ( J_0(\beta a) Y_0(\beta a) + J_1(\beta a) Y_1(\beta a) \bigr ) \end{aligned}$$

with

33 $$\begin{aligned} \int {z \, J_n(a z) \, Y_n(a z) \, \mathrm{d}z}= & {} \frac{z^2}{4} \, \Bigl ( 2 J_n(a z) Y_n(a z) - J_{n-1}(a z)Y_{n+1}(a z)\Bigr )\end{aligned}$$

34 $$\begin{aligned} J_{-n}(z)= & {} (-1)^n J_n(z) \end{aligned}$$

For $Y_0$ or $Y_n$ equations analogous to Eq. (30) or (34) hold, respectively.

## Footnotes

1 In the present example, using as lower bound $T_0 = 0$ K instead of 270 K gives rise to deviations in the 5%-range when the middle of the annealing stage is shifted down to 296 K by reducing *Q* from 1.04 to 0.78 eV.

2 Note: Unlike to the cited paper of Louis and Garcia-Cordovilla [7], here $\alpha$ is defined as the material fraction *not yet* transformed in order to be consistent with the definition of $\alpha$ in “Annealing of vacancies at dislocations” and “Annealing of vacancies at grain boundaries” sections as the fraction of vacancies not yet annealed out.
